# From private office to locker room: how corporate America is transforming academic family medicine

**DOI:** 10.3389/fmed.2026.1819704

**Published:** 2026-05-12

**Authors:** Siegfried O. F. Schmidt, Elizabeth C. Sanders, Frank A. Orlando

**Affiliations:** Department of Community Health and Family Medicine, University of Florida, Gainesville, FL, United States

**Keywords:** autonomy, burnout, industrial engineering, open workspaces, private office, productivity, quality metrics, space utilization

## Introduction

Family medicine (FM) was once synonymous with deep patient connections, continuity of care across generations, and professional autonomy. For academic family physicians (FPs), it also meant a longitudinal mentorship with residents and medical students. Academic FPs represent about 10–20% of all FPs. Residency core faculty and medical school faculty in academic health systems are responsible for the majority of FM teaching of residents and medical students, respectively (1). Today, administrators have grown far faster than physicians, now outnumbering them by 10 to 1 and consuming a large portion of healthcare spending (2–5). As a result, FM has been restructured to include corporate quality dashboards, relative-value units (RVUs), and performance metrics (6, 7); however, these have also impacted FPs' ability to connect with patients and undermines the doctor-patient relationship (7, 8). There is less protected time for education and practice-based research with more clinical obligations tied to institutional productivity metrics (6). Furthermore, the cost to physician practices of dealing with quality measures disproportionately affects primary care and has increased astronomically since 2006, costing 15.1 h per physician per week and $15.4 billion per year (9). The uniqueness of the personal doctor-patient connection is being lost amid a systemic prioritization on patient throughput and revenue generation in the name of euphemisms like access and productivity, respectively (10). This has been further altered by telemedicine where FPs are literally losing touch with their patients.

Physicians have increasingly become blue-collar workers in white coats (11). FM became primary care and the FP a healthcare provider, stripped of meaningful input into administrative decisions shaping clinical care (12, 13). Physicians, especially FPs, are increasingly working in hospital- or private equity–owned organizations rather than physician-owned entities (7, 8, 12–14). The loss of FP autonomy negatively affects timeliness and quality of care, patient satisfaction, physician wellbeing, and attrition (13, 14). What was once a vocation dedicated to caring for patients has quietly been transformed into a production line, with doctors laboring on factory floors while patients are the commodity, their burden of disease quantified for maximum profitability.

## Shift in priorities: the corporatization of family medicine

The migration of physicians can be seen in every community—private practice is an endangered species (14). Hospital corporations and private equity have absorbed and merged many once privately owned FM practices (7, 8, 12–14). Hospital corporations capitalize on the entities that feed emergency rooms and admissions (15). Academic health centers are increasingly run by administrators who have never practiced medicine or have long forgotten what FM was all about.

The Medicare managed care model shifted in the late 1990s with the creation of Medicare Part C (originally Medicare+Choice and now Medicare Advantage) (16, 17). Significant changes to Medicare's interactions with managed care plans dawned a new economy in the commercial health insurance industry, a sector later expanding with the Affordable Care Act (ACA) in 2010. While the ACA created access to health insurance for many Americans, it also created a significant financial opportunity (18, 19). Private equity firms commoditized the Medicare Advantage plans, reaping significant profits in shared savings that are not always shared with frontline FPs (20–22).

To capture these reimbursements, health systems compel FPs to emphasize disease severity/risk and visit complexity because these mean higher payment. Physician-employees maximize revenue for corporate practices by being salaried, assigned a patient panel, and linked to multiple advanced practice providers to optimize RVUs, Hierarchical Condition Coding (HCC), and billing the highest level of Evaluation/Management services allowed (19, 23–26). This did not improve patient outcomes (19) and resulted in abuse. Many high-value HCC diagnoses unsupported by medical record documentation led to billions of dollars of overpayment by Centers for Medicare & Medicaid Services to Medicare Advantage plans (27, 28).

## The evolution of office space

Academic healthcare systems increasingly emulate corporate-owned health systems where individual offices are intentionally consolidated into open or locker room-style workspaces utilized by multiple providers in the name of system integration (29–38). Given the growing employed-physician workforce disproportionately affects primary care (14), FM may be one of the last specialties to still value and retain a private office within clinic. However, when productivity is measured per square foot per provider, it is easy to see corporate motivation to repurpose office space into additional, revenue-generating exam rooms, specialist-integrated space, or administrative offices for staff managing quality measures (9, 34–39).

For full-time FM faculty, losing a personal office is more than an inconvenience; it is a loss of personal identity. FPs generally spend more time in their clinic and office than home whereas other specialties spend substantial time away from their office for procedural or inpatient duties (40, 41). Consequently, the loss of individual offices disproportionately impacts FPs. That private space symbolizes stability, continuity, confidentiality, and mentorship. It is where one has the silence to think and regroup, to make difficult phone calls to patients to share grave results, to call a colleague to discuss complicated patient referrals, or to debrief medical students and residents after a tough encounter. Certainly, a shared desk, or worse locker room, simply cannot deliver that.

Individual offices are not always converted into extra exam rooms to boost profitability. When renovations are not driven by patient–care or educational needs at all, reconfigurations serve corporate objectives, such as centralizing administrative functions, creating revenue-generating flex spaces, or expanding executive suites (8, 32–36, 42). Executive offices have even been shifted into prime locations previously housing central physician workstations inside hospitals (42).

Administrators can see physicians who contest private space within their clinic as being inflexible. However, those advocating for traditional office designs versus open offices are protecting an essential work environment that enables autonomy, continuity, confidentiality, efficient workflow, and mentorship (12, 13). The shift toward locker room–style workstations indicates a broader transformation: corporate America has entered academic medicine, placing financial metrics and space utilization ahead of the traditional academic mission of education through patient centered care (6–8, 12, 13).

While FPs who have been practicing for years may balk at the loss of private offices, new FPs are unlikely to even request private office space in favor of unknowingly accepting the conversion to locker room–style workstations. Contemporary residencies generally enculturate residents into the corporate model of medicine that favors space–utilization and financial metrics ahead of the traditional academic model of patient–centered care and education. This is evident by the fact that younger physicians under age 45 were much less likely to be practice owners in 2022 (31.7%) compared to a decade earlier (44.3%), reflecting changing practice preferences and employment settings among newer physicians (43). New FPs are entering corporate healthcare environments where private offices are less common by default, so the demand is lower or accommodated differently. While employed physicians need to make do with what is offered, new hires may have different expectations than residency as recent graduates are leaving their first job in less than 2 years, whereas physicians have historically stayed in their first job for about 6 years (44).

## What family medicine stands for

FPs were the original general practitioners (45). FM as a specialty was built on a foundation counter to corporate America: relationships, continuity, and community instead of profitability, efficiency, productivity, and compliance. Traditional academic FP offices were always open to patients, colleagues, staff, and students. Visibility and presence define the FP; without them, FM loses its core distinction.

## Rationales for dedicated offices for full-time family physicians in ambulatory clinics

### Clinical rationale

**Figure 1:**
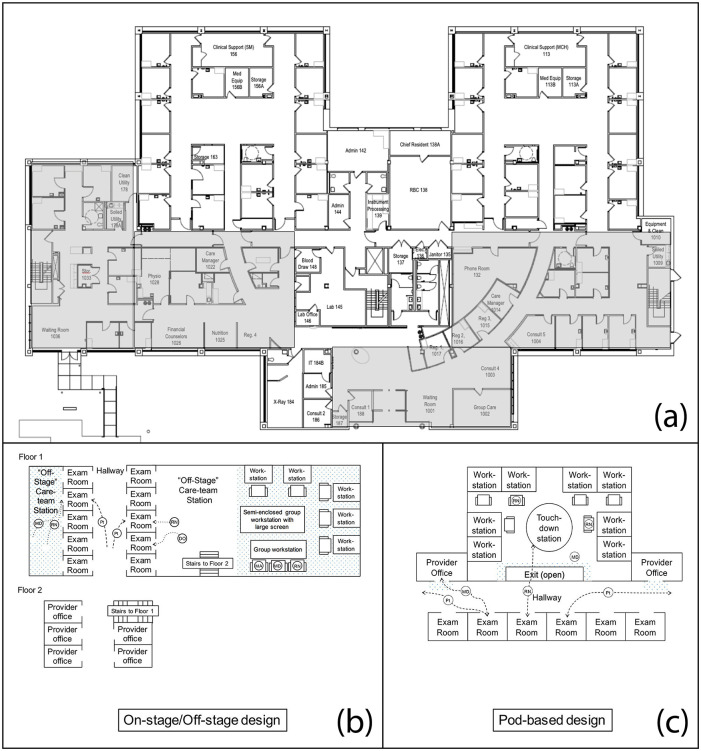
Examples of workspaces. **(a)** Traditional workspace clearly illustrates the distance between exam rooms (within unshaded areas) and offices (within shaded areas), highlighting the resulting usability challenges during clinical workflows [Adapted from source: Koonce T, Neutze D. Improving Patient Care Through Workspace Renovation and Redesign: A Lean Approach. *Fam Med*. 2020; 52(6):435–439. doi: 10.22454/FamMed.2020.429243 (38). Copyright © 2020 Society of Teachers of Family Medicine. Used by permission]; **(b)** On-stage/off-stage workspace shows the office located on another floor, making meaningful use nearly impossible; **(c)** Pod-based workspace demonstrates exam rooms in proximity to offices and care-team station, fostering direct collaboration with nursing while balancing the need for privacy [Adapted (b, c) from source: Karp Z, Kamnetz S, Wietfeldt N, Sinsky C, Molfenter T, Pandhi N. Influence of Environmental Design on Team Interactions Across Three Family Medicine Clinics: Perceptions of Communication, Efficiency, and Privacy. *HERD*. 2019; 12(4):159–173. doi: 10.1177/1937586719834729 (36). Copyright © 2019 SAGE Publications. Reprinted by permission of SAGE Publications].

Physicians' consistent physical presence is essential for continuity of care, same-day access, and prompt decision-making. In-person, clinic availability provides timely evaluation of urgent conditions, ensures follow-up after complex visits, and sustains longitudinal doctor-patient relationships, all of which are core FM principles. Although some advocates of redesigning traditional workspaces argue that decentralized private offices go “unused” (Figure 1a) (38), the open model can *decrease* face-to-face collaboration in favor of electronic messages (33, 46), reducing real-time coordination and oversight. Noise and irrelevant speech created by open architecture degrades clinical performance, and such interruptions or distractions correlate with higher rates of medical errors, particularly for cognitively demanding tasks (47–49). While a major issue with open workspaces is lack of privacy for focused or confidential work, “talking rooms” contribute to stakeholder perception of unnecessary space and do not fully solve this conundrum (34, 50). Designs dividing onstage spaces where patients travel, from offstage spaces where staff can work without patient presence, reflect the traditional practice of providing physician offices distant to open patient zones (Figure 1b) (33, 36).

Rather than remove the private office, which has met significant resistance during planning (35, 51), it is critical for designers to consider the tasks, culture, and technology of an organization to maintain effective communication and care coordination (33). If shared cubicles within open workspaces designed in the 2010's went unused at their inception because their semi-private space was not a functional replacement for the private office (35), this is even more true in the 2020's and beyond. For example, many organizations are using artificial intelligence (AI)-powered ambient dictation, so FPs no longer need a semi-private space for dictation as the bulk of documentation and billing are completed in the exam room (52, 53). However, that is not all the reasoning a patient needs, and most FPs will spend more time thinking in their office before adding their final rationale to the assessment and even adjusting the management plan.

Physicians in open workspaces with shared cubicles tend to stay in the team area when involved in patient care (35), demonstrating that the best workspaces retain the necessary benefits of physician offices nearby exam rooms in combination with care-team stations (pod-based designs), a proximity that fosters direct nursing collaboration (Figure 1c) (36). Clinic layout affects patient flow, timeliness, resource utilization, and ultimately patient satisfaction (54), and adjacent private office space allows physicians to efficiently manage patient flow, review documentation, and supervise team members without interruption (36).

A private office establishes physicians' institutional identity and fosters patient confidence that their physician is part of the clinic community and not merely rotating through it (51). This stability and continuity benefit not only faculty morale but also patient trust, outcomes, and satisfaction (55–57). Patient trust in medicine has gradually dwindled over the years and yet remains an indispensable part of the therapeutic bond with their primary care physician (PCP), directly affecting treatment compliance and overall health outcomes (56, 58). Because Press Ganey patient satisfaction scores can affect reimbursement rates (59, 60), maintaining a private office has tangible financial implications in addition to physician wellbeing implications (61).

### Educational rationale

An academic primary care clinic without private offices cannot function as a center of teaching and mentorship. It is of utmost importance for students and residents to have privacy when they need to seek guidance, review cases, and receive regular feedback. The private office represents professional presence and stability, reinforcing the physician's leadership role and enhancing the educational and team culture of clinic.

### Organizational rationale

Full-time faculty physicians routinely handle patient protected health information, faculty and student evaluations, administrative leadership meetings, medico-legal correspondences, peer-review material (intellectual property), and in some cases even inventions. A private office is vital to maintain HIPAA compliance, support secure electronic and paper documentation, and allow for confidential discussions with other faculty, residents, students, staff, inter-institutional research collaborators, and/or attorneys. Even with various security technologies, shared offices raise legitimate risks of unintended disclosure and breaches of professional boundaries (51).

Research consistently links the physical work environment to physician burnout and professional satisfaction which correlates with physician retention (51, 62–64). Compared with individual offices, shared or open workspaces are not beneficial to employees' health and lead to deleterious effects on staff health, wellbeing, and productivity (65). The process of hiring and onboarding a new physician costs organizations on average $150,000 to $1.2 million (66, 67). In a 2024 cross-sectional study of PCPs, satisfaction with the physical workspace was associated with a 50% reduction in burnout risk (68). Dedicated, ergonomically designed workstations improve concentration, reduce fatigue, improve physician wellbeing, and contribute to recruitment and retention, especially among senior and full-time clinicians anchoring the practice (69, 70).

## Conclusion

As AI tools perform increasingly more medical tasks that challenge the traditional roles of physicians (71), the primary care workforce is transitioning further from a physician-dominated model (72). A personal, on-site office is not a luxury; it is essential for identity and professionalism. Forsaking that standard reduces FPs to easily exchangeable parts in a system that already debases physicians. Physicians become replaceable when autonomy is reduced—if FPs accept the locker room-style, they accept the corporate message that FPs are expendable (45, 57, 62, 73–75).

Assigning each full-time, academic FP an individual office is a clinical, educational, and organizational necessity, especially as AI-driven healthcare technologies continue to advance (52, 53). It directly supports operational efficiency and compliance, patient safety and privacy, physician wellbeing, and quality of care, aligning with institutional missions of excellence in patient care, community service, teaching, and discovery.
